# Foraging Ranges of Insectivorous Bats Shift Relative to Changes in Mosquito Abundance

**DOI:** 10.1371/journal.pone.0064081

**Published:** 2013-05-07

**Authors:** Leroy Gonsalves, Brad Law, Cameron Webb, Vaughan Monamy

**Affiliations:** 1 School of Arts & Sciences, Australian Catholic University, North Sydney, New South Wales, Australia; 2 Forest Science Centre, Department of Primary Industries, Beecroft, New South Wales, Australia; 3 Department of Medical Entomology, Westmead Hospital and University of Sydney, Wentworthville, New South Wales, Australia; University of Regina, Canada

## Abstract

The need to develop effective management strategies for insectivorous bat populations requires an understanding of factors influencing habitat use. Availability of pest prey, such as mosquitoes is likely to be one such factor. To assess whether this is the case, we radio-tracked *Vespadelus vulturnus* Thomas (little forest bat), a predator of *Aedes vigilax* Skuse (saltmarsh mosquito), in saltmarsh and adjacent coastal swamp forest during periods of high and low *Ae. vigilax* abundance. When mosquito abundance in structurally-open saltmarsh was similar to the more cluttered coastal swamp forest, use of saltmarsh by *V. vulturnus* was disproportionately greater than its availability, with saltmarsh selected preferentially for foraging. However, at times of low *Ae. vigilax* abundance in saltmarsh, use of saltmarsh by *V. vulturnus* was reduced and all habitats were used in proportion to availability in the study area. This is the first radio-tracking study to demonstrate a shift in foraging range by an insectivorous bat species correlated with fluctuations in the distribution and abundance of a particular prey resource. The shift in foraging range by *V. vulturnus*, corresponding with a spatio-temporal variation in abundance of *Ae. vigilax* highlights the importance of mosquitoes as a dietary item. Broadscale pest control of *Ae. vigilax* may have ecological implications for the diet and habitat use of *V. vulturnus*. An adaptive management approach is proposed, whereby careful monitoring of insectivorous bat populations is recommended before and after any application of broadscale mosquito control measures. We also suggest a precautionary approach is taken such that broadscale control of mosquitoes avoids the lactation period of bats, a time when their energetic demands are greatest and when there is reduced risk of contracting mosquito-borne diseases transmitted by *Ae. vigilax*.

## Introduction

Conservation of insectivorous bats requires appropriate management of habitats in which bats forage as well as any prey resources these habitats sustain. To assist policy-makers, greater knowledge of factors influencing habitat use by foraging bats is required. While vegetation clutter is known to influence mobility and foraging activity of many bat species [1], [2], [3], availability of prey is also a key factor [4], [5]. Many studies have investigated the influence of prey availability on bat activity using prey abundance as a proxy for prey availability. However, it has been shown that vegetation clutter also can reduce access to these prey resources for bats [5], [6]. This may suggest that habitats with high insect abundances may not always be suitable foraging habitats for bats if acoustic complexity restricts access to abundant prey resources.

Mosquitoes can be abundant locally and have been identified as prey items for many bat species world-wide [7], [8], [9]. *Aedes vigilax* Skuse (saltmarsh mosquito) is an estuarine species that is a serious nuisance biting pest and has been identified as an important vector of mosquito-borne pathogens such as Ross River virus (RRV) and Barmah Forest virus (BFV) [10]. Notwithstanding potentially significant public health risks, this species can cause substantial nuisance-biting impacts and broadscale mosquito control programs have been implemented in many Australian coastal regions [11]. Although there is evidence that *Ae*. *vigilax* may be an important dietary item for insectivorous bats foraging within saltmarshes [12], [13], [14], [15], no study has specifically investigated the importance of the mosquito to insectivorous bats.

Population abundances of *Ae. vigilax* are influenced strongly by tidal and rainfall inundation of larval habitats (i.e., coastal saltmarsh and mangrove communities) and, as a consequence, can be highly variable spatially and temporally. However, general patterns such as peaks in abundances can be predicted [16]. Generally, more abundant populations tend to be present approximately two weeks after inundation of saltmarshes by spring tides and/or heavy rainfall, representing a more plentiful prey resource for insectivorous bats. Given that *Ae. vigilax* can disperse >5 km from larval habitats [17], adjacent coastal swamp forests are likely to provide refuge for this mosquito species as well as providing sources of blood meals, sustaining high abundances for longer periods.

*Vespadelus vulturnus* Thomas (little forest bat) is a small insectivorous bat closely associated with forest and woodland habitats in coastal and inland regions of south-eastern Australia [18]. This species feeds opportunistically on flying insects with a diet composed of locally abundant prey items including moths and beetles, [18]. With a high frequency modulated echolocation call (end frequency 50–53 kHz) capable of detecting small prey items, *V. vulturnus* has been observed hawking (hunting) mosquitoes in estuarine habitats [14]. It also has been suggested [19] that when mosquitoes are abundant, they are likely to represent a major portion of the dipterans present in the diet of *V. vulturnus.* DNA from *Ae. vigilax* was detected in faeces collected from 11 of 20 *V. vulturnus* individuals in an area where *Ae. vigilax* is abundant [20].

Ultrasonic detectors revealed that activity of small, high frequency echolocating bat species (including *V. vulturnus*) was correlated with mosquito abundance [13]. However, detectors only record population level responses. Radio-tracking is needed to document the response of individuals and can determine to what extent they shift foraging ranges. We hypothesised that shifts in foraging range by *V. vulturnus* would correlate closely with spatio-temporal variation in *Ae. vigilax* population abundances in acoustically simple saltmarsh. To test this, we investigated habitat use by *V. vulturnus* over two periods predicted to sustain relatively large and small *Ae. vigilax* population abundances. Habitat use was assessed by radio-tracking *V. vulturnus* in saltmarsh where adult *Ae. vigilax* emerge and in neighbouring, but acoustically more complex, coastal swamp forest that provides a sheltered habitat for host-seeking mosquitoes. At the same time, mosquito populations and other aerial insects were surveyed nightly in both habitats.

## Materials and Methods

### Ethics statement

All work was carried out under scientific licence (S12771) provided by the NSW National Parks and Wildlife Service. Animal ethics permits (TRIM no. 09/7861) for harp trapping and radio-tracking were obtained from the NSW Director-General's Animal Care and Ethics Committee.

### Study site

**Figure 1:**
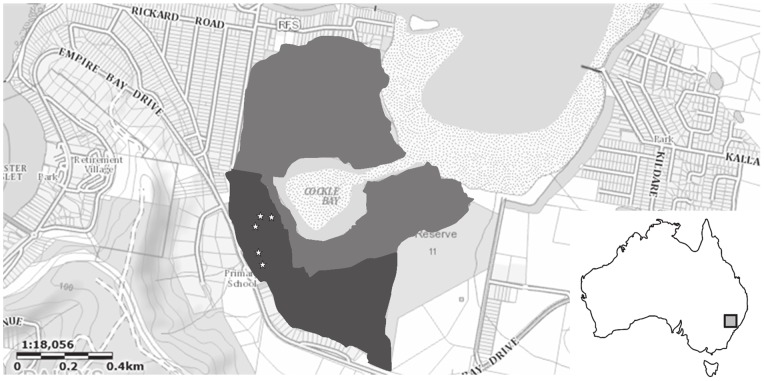
Study area. Map of study area (inset: map of Australia indicating relative location of Empire Bay). Stars represent harp trapping locations within coastal swamp forest habitat (dark grey). Saltmarsh and mangrove habitats (light grey) are visible around Cockle Bay.

The study area was located in the Empire Bay region (33°29′57″S, 151°21′40″E) of the Central Coast of New South Wales, Australia (Fig. 1). This region is approximately 50 km north of Sydney and experiences a warm sub-tropical climate.

Within the study area, a large national park and a number of smaller nature reserves sustain populations of hollow-roosting and cave-roosting insectivorous bats including six species listed under the NSW *Threatened Species Conservation Act* 1995 [21]. The most commonly recorded species in ultrasonic bat detection surveys included *Chalinolobus gouldii* Gray (Gould's wattled bat), *Mormopterus* sp. 2 Peters (eastern freetail bat), and *V. vulturnus*. The latter was selected for this study as it is a small bat (∼4 g) capable of discerning small prey items with its high frequency echolocation call (end frequency 50–53 kHz) and has been found to consume mosquitoes within the study area [20].

Coastal saltmarsh and coastal swamp forest are two threatened vegetation communities (NSW *Threatened Species Conservation Act* 1995) that occur in the area and provide important larval and refuge habitat for many estuarine and freshwater mosquito species including *Ae. vigilax*. Occurring at lower elevations than coastal swamp forest but higher than mangroves [22], saltmarsh is subject to tidal inundations and is dominated by flowering plants, principally low-growing salt-tolerant succulent herbs [23], including *Sarcocornia quinqueflora* (Bunge ex Ung.-Sternb.) A.J.Scott) (samphire) and *Samolus repens* (J.R.Forst. and G.Forst.) Pers) (creeping brookweed). Trees and shrubs are mostly absent. Coastal swamp forest occurs in poorly drained depressions. It has a typical canopy height of 13 m with an overstorey dominated by *Melaleuca quinquenervia* (Cav.) Blake) (broad-leaved paperbark), *Eucalyptus robusta* Anon (fringing swamp mahogany) and *Casuarina glauca* Sieber ex Spreng (swamp oak). It has a dense understorey of wetland or mesic shrubs [24].

One small nature reserve (Cockle Bay) contains approximately 18 ha of coastal saltmarsh and 20 ha of coastal swamp forest. All arthropod sampling was conducted in these two vegetation communities within the nature reserve, while bat trapping was confined to flyways in coastal swamp forest. Approximately 300 m south of the nature reserve is an adjacent up-sloping forest, while low density (2.5 dwellings ha^−^1) residential areas are located 200 m to the east and 1 km west of the nature reserve.

*Harp trapping of bats, attachment of radio-transmitters and radio-tracking methods* Habitat use by *V. vulturnus* was investigated by radio-tracking in two periods during the austral late summer (February 2010) and early autumn (March 2010). Bats were trapped in harp traps along flyways in coastal swamp forest adjacent to saltmarsh. In February 2010, 10 *V. vulturnus* individuals were radio-tracked while six were radio-tracked three weeks later (March 2010), with three individuals tracked in both periods (see Methods S1).

Each trapped individual was fitted with a LB-2N radio-transmitter (Holohil, Carp, Canada), attached between the shoulder blades with Vetbond (3M, Pymble NSW). Each transmitter had an aerial length of 10 cm and weighed 0.31 g, representing 7.95% of *V. vulturnus* mean mass. While the radio-transmitters exceeded the guideline of 5% of body mass suggested by [25], heavier transmitters have been used to study the same species as well as other similar sized bats and have not been reported to restrict the mobility of individual bats significantly [26], [27]. Additionally, pregnant females can weigh 6.5 g and are therefore capable of carrying at least 42% extra body mass.

Estimated locations for bats fitted with transmitters were recorded at least 10 min apart on foot throughout the night by bisecting or triangulating the signal direction (see Methods S1). Location data only were obtained over a maximum of 10 consecutive nights each tracking period as it was predicted that mosquito population abundances after the 10th night would not be consistent with preceding nights due to the time lag from inundation of saltmarsh and the resulting egg hatches. To provide data about distances travelled by bats to foraging sites, day roosts of bats fitted with radio-transmitters were located (see Methods S1).

### Analysis of radio-tracking data

Successful triangulated or bisected locations (see Methods S1) were plotted in ArcMap 9.0 (ESRI, Redlands, USA) and overlayed onto a vegetation layer of the study area [24]. For each tracking period, ≥15 foraging locations (see Methods S1) were used to calculate foraging ranges (95% fixed kernel density estimator (KDE)) in ArcMap 9.0 using the HRT extension [28].

**Figure 2:**
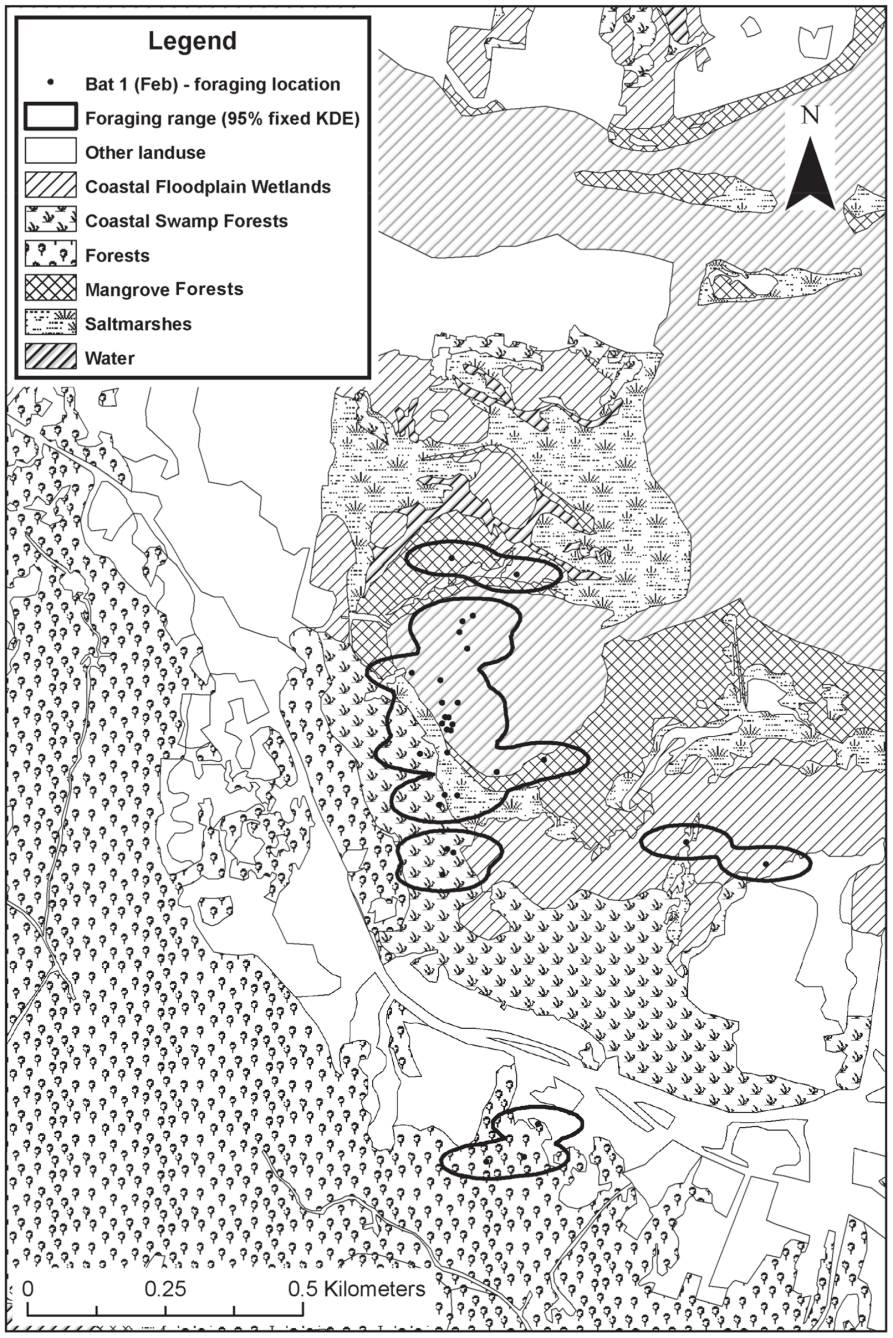
Spread of habitats in study area. Typical GIS output illustrating spread of habitats in the study area and the foraging locations (‘used habitat’) used to construct foraging ranges (‘available habitat’).

Compositional analysis [29] was used to determine if bats were using habitats proportional to availability or whether they preferentially selected habitats, and whether this changed between tracking periods. While the analysis can be undertaken at two scales (population and the individual), roost locations of two individuals tracked in February 2010 were located >1.8 km from roosts of other tracked individuals, suggesting foraging data were likely to be representative of more than one population/social group. Use of foraging data collected from individuals of multiple social groups has the potential to confound foraging preferences at the population scale, since habitats available to members of one social group may not be available to members of another social group. For this reason, compositional analysis only was undertaken at the individual scale. For the analysis, the proportion of bisected/triangulated locations within each habitat was treated as a measure of habitat use, while available habitats were considered to be those that made up an individual's foraging range (95% KDE) (Fig. 2).

Habitats assessed in compositional analyses were coastal floodplain wetlands, coastal swamp forest, mangroves, saltmarsh, open water and a forest complex consisting of wet sclerophyll forest, dry sclerophyll forest, and subtropical rainforest. (Given calculated transmitter error and average error ellipse, it was deemed appropriate to pool these forest habitats together into the one complex).

A chi-squared goodness-of-fit test was used to assess whether habitat selection was non-random and whether each habitat was used in a similar proportion to its availability. Differences between log-transformed relative proportions of both used and available habitats were used to rank habitats according to whether they were being used more than other habitats after accounting for each habitat's availability. A Wilcoxon-pairwise comparison was used to ascertain the significance of these ranks.

### Surveillance of available prey

In each habitat, mosquito abundance was surveyed nightly using two CO_2_ – baited encephalitis virus surveillance (EVS) traps [30] (Australian Entomological Supplies, Bangalow, NSW, Australia), while other flying insects were sampled using a single light trap (Australian Entomological Supplies, Bangalow, NSW, Australia). Traps were set in forest gaps within the coastal swamp forest, while in coastal saltmarsh, traps were set along the interface of the saltmarsh habitat and a stand of encroaching mangroves. Captured mosquitoes were keyed to species [31] and the abundance of each was recorded. All light trap specimens <2 mm in size were pooled together while all other specimens were sorted into three insect orders (Lepidoptera, Coleoptera, Diptera), with any other specimens pooled into an ‘other’ category. Insects then were oven dried at 60°C for a minimum of 48 h and until a constant mass could be recorded (to nearest 1×10^−5^ g) and used as a measure of biomass. Log-linear analysis was used to compare *Ae*. *vigilax* abundance and nightly insect biomass between habitats (saltmarsh/coastal swamp forest) and tracking period (February/March 2010).

## Results

### Prey abundance

In all, 13 243 mosquitoes representing 13 species were collected over both tracking periods (Table 1). *Aedes vigilax* was the most abundant species in each habitat irrespective of tracking period, representing ≈74% of specimens trapped in both habitats during February 2010 and ≈56% and ≈85% of specimens trapped in saltmarsh and coastal swamp forest, respectively during March 2010. Other commonly collected mosquito species were *Ae. alternans* Westwood (Hexham grey), *Culex sitiens* Wiedemann (saltmarsh culex) and *Cx. annulirostris* Skuse (common banded mosquito).

**Table 1:** Mosquito species and their total abundances in saltmarsh and coastal swamp forest during February 2010 and March 2010.

Species	FEBRUARY 2010 (n = 10)		MARCH 2010 (n = 8)	
	Saltmarsh	Coastal swamp forest	Saltmarsh	Coastal swamp forest
*Aedes alternans*	564 (10.29)	56 (1.28)	243 (24.70)	23 (0.96)
*Ae .imperfectus*	16 (0.29)	7 (0.16)		
*Ae. multiplex*	49 (0.89)	219 (4.99)	7 (0.71)	92 (3.83)
*Ae. notoscriptus*	68 (1.24)	49 (1.12)	18 (1.83)	50 (2.08)
*Ae. procax*	46 (0.84)	499 (11.37)	3 (0.30)	24 (1.00)
*Ae. vigilax*	4051 (73.91)	3243 (73.92)	555 (56.40)	2035 (84.83)
*Anopheles annulipes*	6 (0.11)	20 (0.46)	2 (0.20)	12 (0.50)
*Coquillettidia linealis*		2 (0.05)		
*Culex annulirostris*	291 (5.31)	155 (3.53)	48 (4.88)	67 (2.79)
*Cx. molestus*	1 (0.02)	3 (0.07)	2 (0.20)	42 (1.75)
*Cx. qinquefasciatus*	15 (0.27)	8 (0.18)	8 (0.81)	41 (1.71)
*Cx. sitiens*	350 (6.39)	116 (2.64)	98 (9.96)	13 (0.54)
*Verrallina funerea*	24 (0.44)	10 (0.23)		
Total	5481	4387	984	2391

NB. Values in brackets represent percent of total mosquito abundance in each habitat.

**Figure 3:**
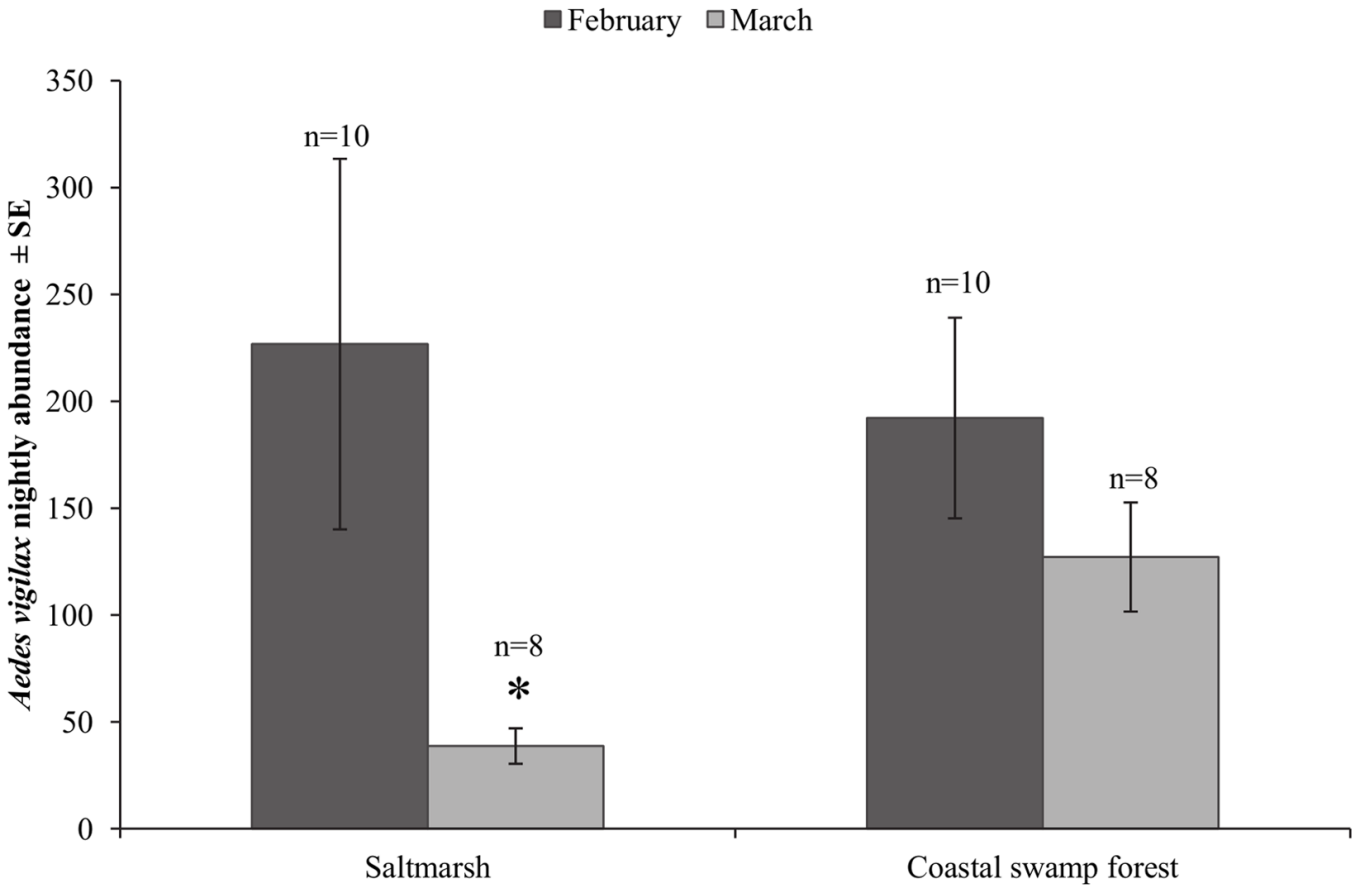
Nightly *Aedes vigilax* abundance. Mean nightly abundance of the *Ae. vigilax* in saltmarsh and coastal swamp forest during February 2010 and March 2010. * Indicates interaction effect.

During February 2010, 4 387 mosquitoes representing all 13 species were trapped in the coastal swamp forest, while 5 481 mosquitoes representing 12 of the 13 species were collected from saltmarsh. In March 2010, 2 391 and 984 mosquitoes representing 10 of the 13 species were sampled in coastal swamp forest and saltmarsh, respectively. Log-linear analysis indicated that nightly *Ae. vigilax* abundance was significantly lower in saltmarsh habitat during March 2010 (L.R. *χ* ^2^(1) = 892.440, P<0.001; Fig. 3).

A total of 37.27 g of insect biomass was collected in light traps over both tracking periods (Table 2). During February 2010, 10.24 g and 11.15 g of insect biomass was collected in light traps in saltmarsh and coastal swamp forest habitats, respectively. In March 2010, 7.72 g and 8.14 g of insect biomass was collected in light traps set out in saltmarsh and coastal swamp forest, respectively. During both tracking periods, lepidopterans and insects <2 mm in size were the two groups that contributed the greatest biomass to saltmarsh light trap collections, while coleopterans and lepidopterans provided the greatest biomass to coastal swamp forest light trap collections.

**Table 2:** Total insect biomass (g) collected in light traps in saltmarsh and coastal swamp forest during February 2010 and March 2010.

Taxa/class	FEBRUARY 2010 (n = 10)		MARCH 2010 (n = 8)	
	Saltmarsh	Coastal swamp forest	Saltmarsh	Coastal swamp forest
Lepidoptera (moths)	4.82 (47.05)	3.63 (32.51)	4.01 (51.96)	4.71 (57.88)
Coleoptera (beetles)	1.27 (12.43)	5.46 (48.94)	0.25 (3.20)	2.07 (25.44)
Diptera (flies)	0.43 (4.24)	0.19 (1.75)	0.15 (1.94)	0.22 (2.71)
Other	0.69 (6.73)	0.81 (7.26)	0.20 (2.59)	0.38 (4.67)
<2 mm	3.03 (29.54)	1.06 (9.55)	3.11 (40.31)	0.76 (9.29)
Total	10.24	11.15	7.72	8.14

NB. Values in brackets represent percent of total insect biomass in each habitat.

**Figure 4:**
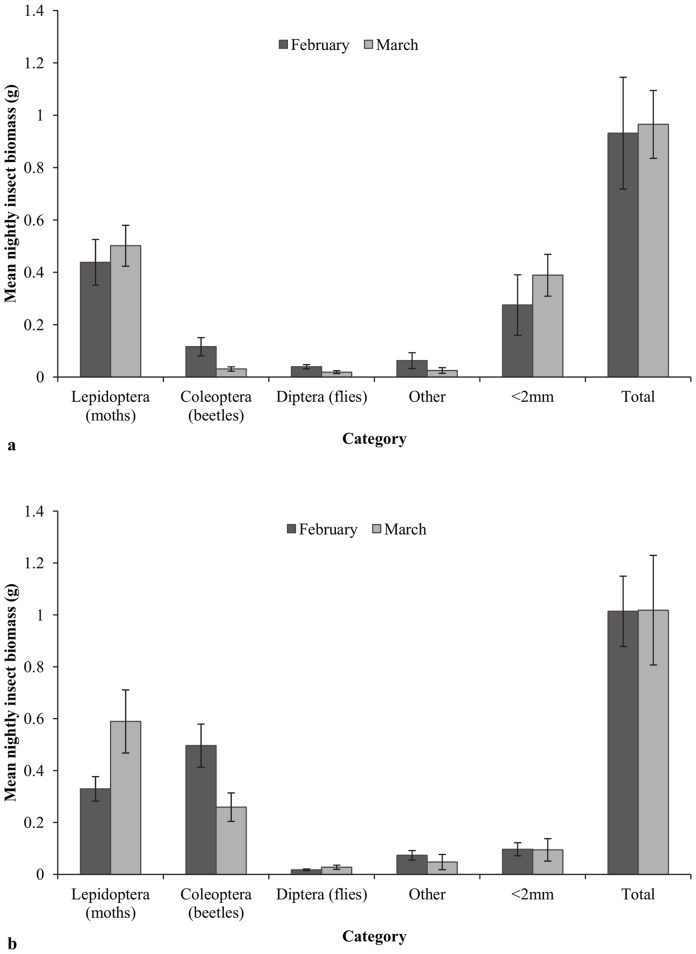
Nightly insect biomass. Mean nightly insect biomass and insect biomass separated by taxa or size during February 2010 and March 2010 in: a) saltmarsh and b) coastal swamp forest. Error bars represent ±1 standard error from the mean.

Mean nightly insect biomass did not differ significantly between habitats or tracking periods (L.R. *χ* ^2^(3) = 0.874, P = 0.832), with 0.93 g and 1.01 g collected from saltmarsh and coastal swamp forest, respectively in February 2010, and 0.97 g and 1.02 g collected in March 2010 (Figs. 4a & 4b). The biomass of lepidopterans, dipterans, other insects and insects <2 mm in size did not differ significantly between tracking periods or habitats (L.R. *χ* ^2^(1) = 0.019, P = 0.991; L.R. *χ* ^2^(1) = 0.097, P = 0.953; L.R. *χ* ^2^(1) = 0.463, P = 0.793; L.R. *χ* ^2^(1) = 2.479, P = 0.290; Figs. 4a & 4b). Coleopteran biomass, however, was significantly greater in coastal swamp forest (L.R. *χ* ^2^(1) = 6.597, P = 0.037; Figs. 4a & 4b).

### Radio-tracking of, and habitat use by, *Vespadelus vulturnus*

In all, 422 triangulation attempts were undertaken for 10 bats during February 2010. Of these, 188 (45%) were successful (at least two triangulated bearings intersected one another). Bats were tracked for a mean of 6.50±2.95 (±1 SE) nights per bat, with 42±7 triangulation attempts, of which 45±7% were successful. Bat #2 and Bat #10 only were tracked for one and two nights, respectively. An active signal from Bat #2 was only detected for one hour after the release of this bat suggesting that the radio-transmitter had probably been removed by the bat. An active signal for Bat #10 was still present on the last night of the tracking session, but only two nights of foraging data were collected since this bat only had been trapped on the penultimate night. Foraging ranges were not calculated for these two individuals.

During March 2010, 327 triangulation attempts were made with 149 of these successful (46%). Bats were tracked for a mean of 5.67±2.25 nights per bat, with 55±11 triangulation attempts, of which 46±5% were successful. Bat #6 was trapped on the penultimate night of the tracking session and only tracked for two nights. A foraging range was not calculated for this individual.

**Figure 5:**
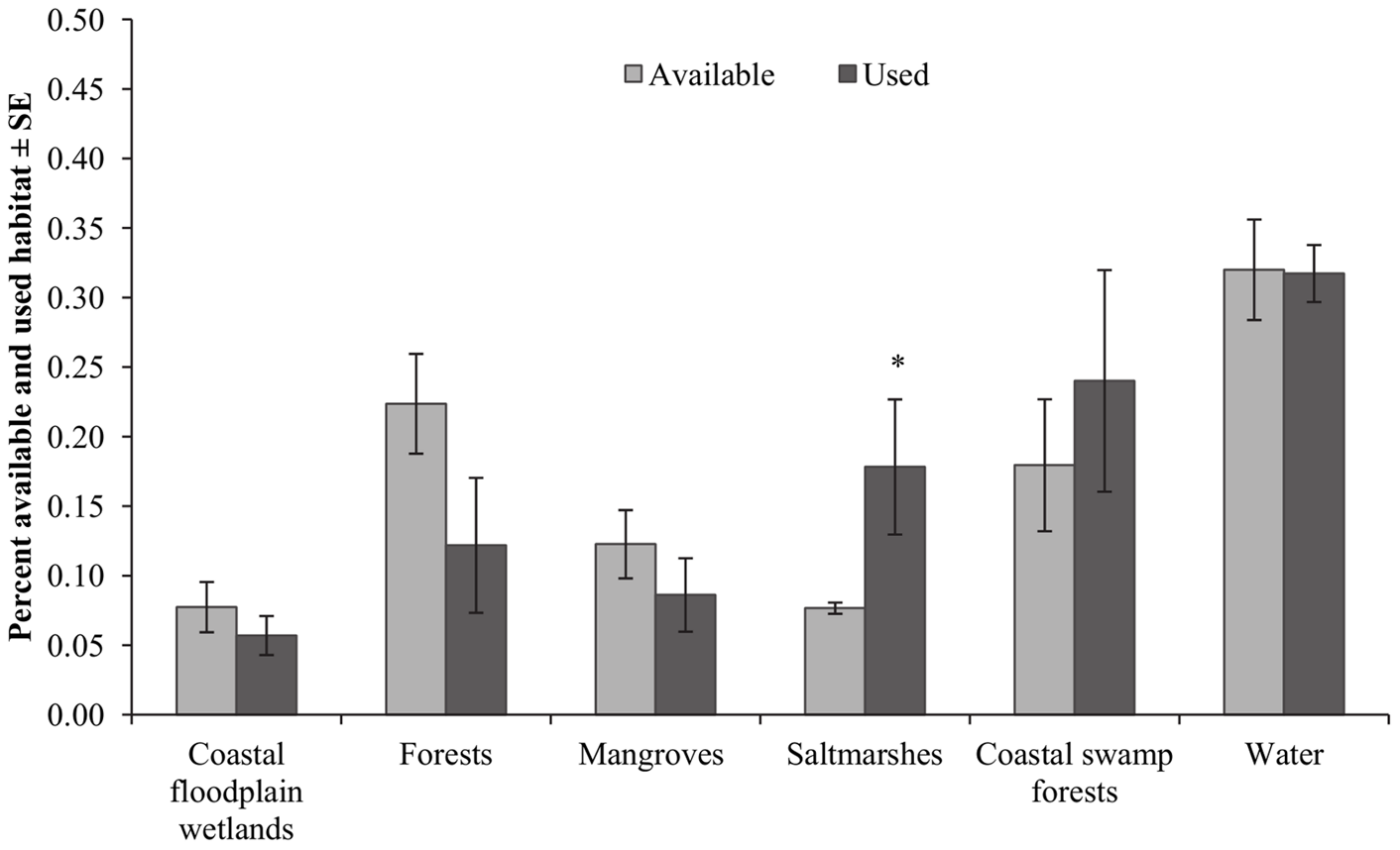
Habitat availability and use in February 2010. Percentage of available and used habitat in February 2010.

Foraging ranges (95% KDE) of *V. vulturnus* individuals were larger in February 2010 (35±4 ha) than in March 2010 (14±7 ha). Habitat use by *V. vulturnus* individuals was non-random in both months (February 2010 – *χ* ^2^(5) = 28.802, P<0.001; March 2010 – *χ* ^2^(5) = 56.480, P<0.001). In February 2010, use of saltmarsh was significantly greater than availability of the habitat (*χ* ^2^(1) = 3.846, P = 0.05), while all other habitats were used in similar proportions to their availability (Fig. 5). Compositional analysis revealed that saltmarsh ranked highest of all habitats followed by coastal swamp forest, coastal floodplain wetlands, open water, the forest complex and mangrove forests (Table 3). Use of saltmarsh was significantly greater than use of coastal floodplain wetlands (Z = −2.380, P = 0.017), mangrove forests (Z = −1.960, P = 0.050) and the forest complex (Z = −2.380, P = 0.017).

**Table 3:** Ranking matrices for *V. vulturnus* in February 2010 and March 2010, based on comparisons of the proportions of locations for each bat in each habitat type with the proportion of each habitat type available within the bat's foraging range (95% KDE).

Habitat	Coastal floodplain wetlands	Coastal swamp forests	Forest complex	Mangrove forests	Saltmarsh	Open water	Rank
*February 2010*							
**Coastal floodplain wetlands**	0	−1.06	0.21	0.39	−1.38	−0.17	3
**Coastal swamp forest**	1.06	0	1.27	1.45	−0.32	0.90	2
**Forest complex**	−0.21	−1.27	0	0.18	−1.59	−0.37	5
**Mangrove forests**	−0.39	−1.45	−0.18	0	−1.77	−0.56	6
**Saltmarsh**	1.38	0.32	1.59	1.77	0	1.21	1
**Open water**	0.17	−0.90	0.37	0.56	−1.21	0	4
*March 2010*							
**Coastal floodplain wetlands**	0	−1.43	−0.96	−1.37	−0.96	−1.59	6
**Coastal swamp forest**	1.43	0	0.47	0.06	0.46	−0.16	2
**Forest complex**	0.96	−0.47	0	−0.41	0.00	−0.63	5
**Mangrove forests**	1.37	−0.06	0.41	0	0.40	−0.22	3
**Saltmarsh**	0.96	−0.46	0.00	−0.40	0	−0.63	4
**Open water**	1.59	0.16	0.63	0.22	0.63	0	1

**Figure 6:**
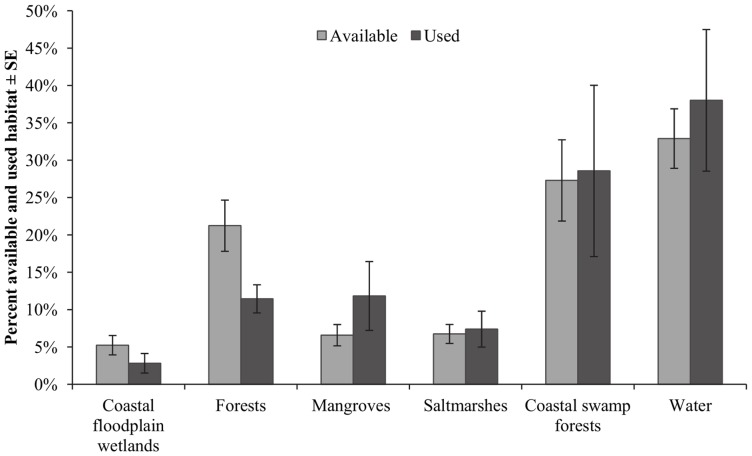
Habitat availability and use in March 2010. Percentage of available and used habitat in March 2010.

In March 2010, all habitats were used in similar proportions to their availability (Fig. 6). Compositional analysis revealed that open water ranked highest of all habitats used by *V. vulturnus* individuals, followed by coastal swamp forest, mangrove forests, saltmarsh, the forest complex and coastal floodplain wetlands (Table 3). The use of open water was significantly higher than the use of the forest complex (Z = −2.023, P = 0.043).

With the exception of one night in March 2010, all radio-tracked bats roosted outside the coastal swamp forest. Most individuals roosted in eucalypt vegetation on an escarpment 300–400 m away adjacent to the coastal swamp forest. Additionally, triangulated bearings for these individuals indicated that these roosts were located <200 m from each other. Three individuals (two in February 2010 and one in March 2010) roosted beneath the metal caps of telegraph poles in urban areas. The two individuals in February 2010 (male and female) only roosted on telegraph poles, roosting on three poles separated by a maximum distance of 670 m. These roost locations were 1.82±0.16 km from the site of capture, while the roost location for all other bats was <600 m from the site of capture.

## Discussion

This is the first study to identify a short-term shift in foraging range by an insectivorous bat species correlated with fluctuations in distribution and abundance of a particular prey resource. The shift in foraging range by *V. vulturnus* corresponded to a change in the distribution and abundance of *Ae. vigilax*, an abundant prey resource for small insectivorous bats. This has implications for the use of broadscale mosquito control to reduce the abundance of *Ae. vigilax*, a recognised vector of mosquito-borne pathogens and a nuisance biting pest [10], [32].

### Prey biomass

*Aedes vigilax* was the most abundant mosquito species in saltmarsh and coastal swamp forest during February and March 2010. This trend has been observed during long-term mosquito surveillance in the study area with *Ae. vigilax* representing 41.2% of all mosquitoes trapped over nine consecutive trapping seasons (unpublished data – L. Gonsalves and C. Webb). While population abundances of this mosquito species can be variable, its consistent presence in the study area provides *V. vulturnus* and other small-sized bats with a reliable prey resource during the austral summer.

As expected, the abundance of *Ae. vigilax* during March 2010 was significantly lower in saltmarsh than in February 2010. This result was in line with expectations as the lack of extensive tidal flooding of saltmarsh in the weeks preceding the March 2010 radio-tracking period did not provide suitable conditions for mosquito development [33]. However, there was no significant difference in the mean abundance of *Ae. vigilax* in coastal swamp forest between the two radio-tracking periods. Mark-release-recapture experiments have shown that *Ae. vigilax* can disperse >5 km from larval habitats [17] and coastal swamp forest is likely to provide a humid refuge as well as a source of blood meals for this species, sustaining high population abundances for longer periods than exposed saltmarsh environments.

Since the trapping techniques used to survey mosquitoes and other insects were not the same, any comparison of mosquito abundance with abundance of other insects must be interpreted with caution. The mosquito traps collect a subset of the extant mosquito population as over a short range they specifically target host-seeking female mosquitoes as those mosquitoes are most attracted to the carbon dioxide-baited traps. A comparison of *Ae. vigilax* biomass with the biomass of other insect fauna revealed that in February 2010, *Ae. vigilax* biomass in saltmarsh (8.02 g) and coastal swamp forest (6.42 g) was similar to the biomass of all other insect taxa combined (assuming one adult mosquito weighs 0.00198 g). In March 2010, *Ae. vigilax* biomass in saltmarsh (1.10 g) and coastal swamp forest (4.02 g) was within the range of biomass contributed by other aerial nocturnal insect fauna. This emphasises the potentially important contribution of *Ae. vigilax* as a food source for *V. vulturnus*.

While average nightly insect biomass did not differ between habitats or tracking periods, the biomass of particular taxa did. Coleopteran biomass was significantly greater in coastal swamp forest than in saltmarsh during both radio-tracking periods. While few studies investigating distribution of beetles in saltmarsh [34], [35] have identified elevation and associated saltmarsh vegetation gradients as variables closely associated with beetle distribution, no studies have specifically investigated beetle distribution along a saltmarsh-adjacent forest gradient. Other studies investigating beetle distribution and abundance in forested areas and more open habitats have reported higher abundances of coleopterans in forests and forest fragments than in adjacent clear cuts [36], forest clearings [37], and open pastures [38]. With relatively few comparative studies of beetle abundances in different structural vegetation associations, it is difficult to compare our results directly to previous investigations.

### Habitat use by *Vespadelus vulturnus*

Given the error associated with radio-tracking, it is often difficult to elucidate habitat use at fine spatial scales and this may result in the use of particular habitats being under- or over-estimated. Many of the foraging locations classified as open water in this study were located close to mangroves that fringe saltmarsh on the seaward side. While some individuals were recorded to commute (>1.8 km) across open water from roosts to foraging areas, it is quite possible that some of these locations were in fact in saltmarsh edge zones (saltmarsh-mangrove interface), where ultrasonic detectors have found bats to be more active than in the interior of the saltmarsh [39]. Additionally, light-tagged *V. vulturnus* individuals released in the saltmarsh interior have been observed commuting to edge vegetation before leaving saltmarsh [20], further supporting the view that these vegetation interfaces provide an edge for bats to forage along. Despite the potential for underestimation of small portions of habitat, compositional analysis revealed that after accounting for availability of habitats within the foraging range, saltmarsh was the most preferred habitat for foraging *V. vulturnus* individuals in February 2010. While *V. vulturnus* has been recorded echolocating and feeding in saltmarsh previously [13], [39], this is the first study to identify the preferential use of saltmarsh for foraging by an insectivorous bat species. *Chalinolobus gouldii* was the species most commonly recorded by ultrasonic detectors in saltmarsh [40], yet radio-tracking of this medium-sized bat (14 g) with a low echolocation frequency (29 kHz), revealed that saltmarsh was used in proportion to its availability [40]. In March 2010, use of saltmarsh by *V*. *vulturnus* decreased, with greater use of open water and coastal swamp forest. However, continued use of both threatened vegetation communities reaffirms that they are important foraging patches for *V. vulturnus*.

### Relationships between prey biomass and habitat use

If prey abundance is influencing habitat use by foraging *V. vulturnus* individuals, one would expect that a change in prey abundance in one habitat from February 2010 to March 2010 would also be reflected in a shift in foraging range over this time. During this study, while *Ae. vigilax* populations were abundant in both saltmarsh and coastal swamp forest in February 2010, *V. vulturnus* preferentially foraged in saltmarsh. However, when the abundance of *Ae. vigilax* was significantly lower in saltmarsh, habitat use by foraging *V. vulturnus* individuals shifted with greater use of coastal swamp forest. This trend was not observed for any other prey taxa measured in this study.

While prey may be abundant in a given habitat, the ability of bats to access these resources can be inhibited by other physical characteristics of the habitat such as vegetation clutter, indicating that prey abundance does not necessarily equate to availability [5], [6]. Preferential use of saltmarsh for foraging in February 2010 may reflect this principle – it is energetically less demanding and perhaps more efficient to locate prey in an open habitat such as saltmarsh than in a cluttered forest environment [41].

A shift to foraging in coastal swamp forest in March 2010 when *Ae. vigilax* was more abundant in this habitat than the neighbouring saltmarsh may indicate that *V. vulturnus* preferentially seeks *Ae. vigilax* as a dietary resource. Alternatively, use of coastal swamp forest by *V. vulturnus* suggests that this species chooses to forage in a habitat that, while being energetically more demanding due to clutter, sustains prey items that are energetically more profitable, mitigating the cost of foraging in this clutter. Lepidopterans and coleopterans contributed the greatest amount of biomass in coastal swamp forest in March 2010. Lepidopterans and coleopterans provide about 25.5 kJ g^−1^ [42] and 21.3 kJ g^−1^ [43] of energy, respectively. Mosquitoes, however, provide lower levels of energy to predators, representing 6.3–14.8 kJ g^−1^ [44]. However, the ‘hardness’ of prey items also will influence the net energy gained from ‘energy rich’ prey items that may require more extensive processing times (mastication and digestion) and thus increased energy expenditure [45]. Handling time (time taken to capture prey) associated with each prey item, presumably, will also influence the habitats in which bats choose to forage [46].

It is possible that other factors may be influencing which habitats *V. vulturnus* selects for foraging. An artefact of the design of this study, based around tidal activity, is the potential influence of lunar illumination on habitat use by *V. vulturnus*. During the March 2010 radio-tracking period (commencing with a waxing crescent moon phase and concluding on a full moon), the level of lunar illumination was greater than the February 2010 radio-tracking period (commencing on the night of a new moon and concluding on the night following the first quarter moon phase). It is possible that *V. vulturnus* foraged in the more sheltered coastal swamp forest during March 2010 to mitigate the risk of predation associated with foraging in open habitats [47]. However, we did not observe any nocturnal predators (e.g., owls) while radio-tracking (L. Gonsalves pers. obs.).

With the exception of one roost, bats were roosting outside the confines of the coastal swamp forest, sometimes >1.8 km away and separated by a water body. Despite this, all radio-tracked individuals were trapped in the coastal swamp forest and foraged there or in the neighbouring saltmarsh each night of the study, further highlighting that these two threatened vegetation communities are important foraging areas for *V. vulturnus*, at least in the study area. Individuals travelled distances greater than previously reported for this species (1370 m from trapping location to roost: [27]), with some individuals travelling >1.8 km from roosts to foraging habitats. Foraging ranges observed during this study were also greater than predicted in a banding study [48] and foraging ranges in forest estimated for the similar-sized *V. pumilus* Gray (eastern forest bat) [26].

### Implications for broadscale mosquito control

Appropriate management of *Ae. vigilax* populations requires consideration of the potential impacts of broadscale mosquito control on the diets of insectivorous bats. A threatening process for bats worldwide is the loss or reduction of prey items due to pesticide use [49]. However, the impact of pesticides on local insect populations is dependent on the type and delivery method of those insecticides. The most commonly used mosquito control agents in Australia (e.g., *s*-methoprene and *Bacillus thuringiensis israelensis*) are generally mosquito-specific and target the aquatic immature stages [11]. While broadscale insecticide use against mosquito populations in Australia is generally only undertaken during periods of epidemic disease activity, use of early season treatment to assist in suppression of irruptions of mosquito populations later in the season is gaining acceptance by authorities undertaking control programs [50]. Given that such control programs can substantially diminish larval mosquito populations, in some cases reducing larval populations by up to 98.2% [50], use of broadscale mosquito control will diminish the important prey resource that *Ae. vigilax* represents to *V. vulturnus* [20]. Use of such mosquito control measures has indirectly been linked to bird declines [32], while declines in bat populations have previously been attributed to deteriorating feeding conditions [51].

### Conclusions

Our study demonstrates a short-term shift in foraging range by *V. vulturnus* correlated with fluctuations in *Ae. vigilax* distribution and abundance, indicating that this mosquito is an important dietary resource for this bat species. Consequently, appropriate management of *Ae. vigilax* populations requires consideration of potential impacts of broadscale mosquito control on the diet of at least one insectivorous bat species. To assess the impact of mosquito control on insectivorous bats more adequately, an adaptive management process should be followed where careful monitoring of bats before and after an application of broadscale mosquito control is required. However, in the interim, control programs should avoid the lactation period of bats, when energetic demands are greatest [52], and risk of contracting mosquito borne disease is reduced [53].

## Supporting Information

Methods S1 Methods used for radio-tracking and calculation of foraging ranges.
(DOCX)
