# Supplementary material for: Foraging Ranges of Insectivorous Bats Shift Relative to Changes in Mosquito Abundance
Source: PLoS One. 2013 May 7;8(5):e64081. doi: 10.1371/journal.pone.0064081 (PMC3646781; doi:10.1371/journal.pone.0064081)
Supplement: Methods S1 — Methods used for radio-tracking and calculation of foraging ranges. (DOCX) [file pone.0064081.s001.docx]

**Supporting Information – Methods S1**

**Methods**

### *Harp trapping of bats, attachment of radio-transmitters and radio-tracking methods*

The sex ratio of bats tracked during February 2010 was 3:7 (M:F), while in March 2010 there were equal numbers of males and females. Although individuals were not marked to differentiate between them, physical characteristics such as forearm length, reproductive condition and sex were used to determine the likely identities of three bats trapped in March 2010 that had been tracked previously (as determined by the presence of a bare patch in the fur where a transmitter had been removed). In February 2010, up to nine bats were tracked simultaneously, while in March 2010 up to five bats were tracked at the same time.

Signals from attached radio-transmitters were located with Australis 26k receivers (Titley Electronics, Ballina, Australia) in conjunction with three element AY/C yagi antennae (Titley Electronics, Ballina, Australia) each night. Simultaneous bearings were taken using Suunto KB-14/360R sighting compasses (Vantaa, Finland) from stations for which GPS locations were recorded. To co-ordinate the taking of simultaneous bearings, tracking teams were in radio contact and one team instructed the other(s) when a bearing was to be taken. Bearings were recorded along with other observations regarding the strength and the consistency of the signal (an indicator as to whether or not the bat was in flight or stationary).

Since bats have been found to be more active in the hours immediately after dusk [1], most survey effort was employed in the 4-5 hours after dusk each night during both tracking periods. However, all individuals also were tracked during the second half of the night (the hours preceding dawn) for at least two nights during each tracking period.

Day roosts of bats fitted with radio-transmitters were located by homing in on signals of stationary bats. When it was not possible to locate a roosting structure (if on private land or in difficult terrain) a series of bearings was taken around the general roost location and later triangulated to provide co-ordinates for the general roost location.

### *Analysis of radio-tracking data*

Simultaneous bearings recorded in the field were entered into LocateIII [[2](#_ENREF_55)] along with their corresponding GPS co-ordinates and were triangulated or bisected. Prior to the start of radio-tracking, transmitters were placed in known locations in saltmarsh and coastal swamp forest to determine a minimum range and error associated with transmitters in each habitat. The distance between triangulated and true locations of transmitters was 78.0±11 m (n=2) in saltmarsh and 94.0±10.9 m (n=3) in coastal swamp forest. Error ellipses of all triangulations (347±175 m^2^; n=15) were smaller than the size of both habitats and were included in all analyses. To determine whether locations calculated using bisects were consistent with those obtained through triangulation, and therefore appropriate to include in foraging analyses, the distance between each triangulated location and the bisected location that resulted from the removal of one bearing from the corresponding triangulation, was measured. This was repeated so that each bearing in a triangulation had been removed once. On nine occasions, the removal of a bearing resulted in an unsuccessful bisection. The average distance between bisected locations and the triangulated location was 73.0±37.0 m (n=36). Since this value was less than the calculated transmitter error in each habitat, all bisects were also included in all analyses.

To determine if sufficient location data were collected to calculate foraging ranges for each individual bat, bootstrap analysis was conducted using the animal movements’ extension [[3](#_ENREF_56)] in ArcView 3.2 (ESRI, Redlands, California, USA). A minimum of 15 foraging locations was deemed a sufficient sample size to estimate foraging range since individuals required 17.7±1.5 foraging locations (range = 15-25) before asymptotes for home ranges were observed. Foraging ranges were not calculated for individuals with < 15 foraging locations. For four individuals in February 2010 with location data that were not deemed to be independent (Schoener’s index <1.6 or >2.4 and/or Swihart and Slade index >0.6), location data (3.0±0.7 locations) were randomly deleted until the indices were no longer significant, as suggested by [[4](#_ENREF_57)]. The Least Squares Cross Validation (LSCV) smoothing parameter (h) was used to determine the spread of the kernel centred over each observation.

The “join” application in ArcMap 9.0 combined the vegetation layer with the foraging range and the bisected/triangulated locations of each individual. The areas represented by used and available habitats were calculated using queries that tallied the area of each habitat within a foraging range and the number of locations in each habitat.

**References**

1. Law BS, Anderson J, Chidel M (1998) A bat survey in State Forests on the south-west slopes of New South Wales with suggestions of improvements for future surveys. Aust. Zool. 30: 467-479.

2. Nams VO (2006) Locate III User's Guide. Nova Scotia: Pacer Computer Software.

3. Hooge PN, Eichenlaub B (1997) Animal movement extension to arcview. Version 1.1. Anchorage: Alaska Science Center - Biological Science Office, U.S. Geological Survey.

4. Ackerman BB, Leban FA, Samuel MD, Garton EO (1990) User’s manual for program HOME RANGE. Idaho: Forest, Wildlife and Range Experiment Station, University of Idaho.
